# Fermentation of Cucumber Extract with Hydromagnesite as a Neutralizing Agent to Produce an Ingredient for Dermal Magnesium Products

**DOI:** 10.3390/ma12101701

**Published:** 2019-05-25

**Authors:** Van Khanh Nguyen, Tam Tran, Tony Crimmins, Van-Tri Luong, Ho Young Kang

**Affiliations:** 1 Department of Microbiology, Pusan National University, Busan 46241, Korea; 2 EcoMag Ltd., Chatswood, NSW 2067, Australia; tam@ecomagnesium.com (T.T.); t.crimmins@abundantproduce.com (T.C.); vantri.luong@unsw.edu.au (V.-T.L.); 3 Abundant Natural Health Pty, Abundant Produce Ltd., Chatswood, NSW 2067, Australia; 4 Particles and Catalysis Research Group, School of Chemical Engineering, University of New South Wales, Sydney, NSW 2052, Australia

**Keywords:** lactic acid bacteria, cucumber extract, magnesium, fermentation

## Abstract

Magnesium is an essential element involved in various biochemical processes in the human body. In addition to oral supplementation, topical magnesium application is another conventional form of magnesium delivery for the treatment of skin diseases and muscle inflammation. Cucumber extract is a well-known superfood for human skin. It has been widely used in various skincare product lines because of its known benefits to the skin. The benefit of cucumber extract to the human skin would be significantly enhanced if the cucumber extract was fermented to convert the reducing sugars to beneficial organic acids. In this study, we developed a protocol for lactic acid fermentation of cucumber extract using hydromagnesite as a neutralizing agent. Various lactic acid bacteria were screened for fermentation of cucumber extract. The best fermenting performance was observed with *Lactobacillus paracasei,* which could convert approximately 13 g/L of reducing sugars (glucose and fructose) to lactic acid and a minor amount of acetic acid within 2 days of incubation. The final fermented cucumber extract contains magnesium in the form of salts of organic acids, which have high absorption ability and bioavailability. The product is a potent ingredient for producing dermal magnesium products.

## 1. Introduction

Natural antioxidants extracted from plants and fruits have attracted much attention from scientists and cosmetics manufacturers. Many of these antioxidants have been used in making skincare formulations and have contributed to skin rejuvenation [1]. Cucumber extracts, which are rich in vitamins, such as vitamin A and C, and antioxidants, have been proven to have various effects on the skin, such as soothing of irritated skin, moisturization, anti-inflammatory, sebum secretion inhibitory, and melanin synthesis inhibitory effects [2,3,4]. As a superfood for the skin, cucumber extract has been added to various skin care products, such as toner, lotion, and cream. However, it also contains a significant amount of sugars, such as glucose and fructose [5,6], which do not provide any benefit to the skin. These sugars are a potential source of fermentation to produce lactic acid, which is a potent compound in skin disease treatment [7]. Lactic acid fermentation of cucumber extract is usually hindered by its low pH, caused by the production of lactic acid [6]. A neutralizing agent is typically required to adjust the pH of cucumber extract to favor the conversion of sugars to lactic acid. The common neutralizing agents used in lactic acid fermentation, such as NaOH, Na_2_CO_3_, KOH, NH_4_OH, Ca(OH)_2_, and CaCO_3_, enrich the final fermented cucumber extract in Na^+^, K^+^, NH_4_^+^, and Ca^2+^, which do not provide any extra value in terms of skincare.

Magnesium is a metal element defined by the symbol Mg and belongs to Group 2 (alkaline earth metals) of the periodic table. Mg is a mineral crucial for human health. It plays a vital role in controlling blood pressure, ensuring normal nerve and muscle functions, and supporting psychological functions and energy production [8,9]. Chronic Mg deficiency could result in many disorders, such as cardiovascular diseases, asthma, diabetes, and osteoporosis [9,10,11]. Suboptimal Mg intake has been recorded in broad sections of the population in many industrialized countries owing to changes in food preparation and nutritive behavior [12]. Oral supplementation or dietary intake are the general ways to overcome Mg deficiency and to ameliorate inflammatory disorders. However, topical Mg application through therapies that have been used for several centuries has been proven to be effective in treating skin diseases [13]. A previous study indicated that hair follicles significantly contributed to the permeation of topically applied Mg through the human skin [14]. In addition, the efficacy of Mg supplementation generally depends on the bioavailability of Mg forms. Organic acid-binding forms of Mg, such as Mg lactate, Mg citrate, and Mg gluconate, are more bioavailable than inorganic Mg salts [8,9,15,16].

This study aimed to develop a potent ingredient for skincare purposes based on the combination of cucumber extract and Mg in a unique product through lactic acid fermentation. This scheme took advantages of fermenting the cucumber extract rich in citric acid to convert available sugars to lactic acid. Hydrated magnesium carbonate, the familiar form of Mg that can be produced at high purity [17], was used as the neutralizing agent in fermentation. Through this process, the Mg ion was dissociated from hydrated magnesium carbonate to bind to organic acids in the fermented cucumber extract. Sugars were depleted and replaced by lactic acid in the fermented solution. The organic Mg salts in the fermented cucumber extract are considered to have higher bioavailability and can be replaced by the inorganic MgCl_2_, which is commonly present in dermal Mg products.

## 2. Materials and Methods

### 2.1. Cucumber Extract and Pasteurization

Cucumber (*Cucumis sativus*) extract is the fluid surrounding the active cucumber seed. The cucumber was organically bred in Cobbitty, New South Wale, Australia. Cucumber extract was introduced to pasteurization right after harvesting by heating to kill the indigenous bacteria and contaminating bacteria during the harvesting process. Then, it was stored in a sterile container and kept in a freezer at −20 °C for the following experiments. 

The pasteurization of cucumber extract was tested at a temperature range of 63 to 121 °C for 1 to 20 min to identify the lowest temperature and shortest time in which all bacteria are killed, and the physicochemical properties of cucumber extract were maintained optimally. The efficacy of pasteurization was determined through bacterial enumeration on an agar plate. Both heat-treated and non-treated samples were serially diluted using phosphate buffer solution, pH 7.2 (34 g/L of KH_2_PO_4_), and an aliquot of 0.2 mL was spread on yeast extract–eptone–glucose (YEPG) agar plate [18,19]. The agar plates were then incubated in an incubator at 25 °C. A colony-forming unit (CFU) was calculated after 3 days of incubation. The chemical ingredients with high purity (>99%) were bought from Sigma Aldrich (Merck KGaA, Darmstadt, Germany).

### 2.2. Hydromagnesite

Highly pure hydromagnesite or hydrated magnesium carbonate (HMC) is the product of magnesium recovered from bittern or brine [17]. HMC (>99%) was obtained from EcoMag Ltd. (Chatswood, NSW, Australia). Fine powder of HMC was re-slurried with water at a ratio of 1:3 (weight/weight) before being used for neutralization of cucumber extract.

### 2.3. Lactic Acid Bacteria

A total of 9 obligately homofermentative and facultatively heterofermentative lactic acid bacteria were used in this experiment. *Lactobacillus acidophilus* KCTC 3164, *Lactobacillus delbrueckii* subsp. *lactis* KCTC 3636, *Lactobacillus salivarius* subsp. *salicinius* KCTC 3600, *Lactobacillus helveticus* KCTC 3545, *Lactobacillus casei* KCTC 13086, *Lactobacillus paracasei* KCTC 13169, *Lactococcus lactis* subsp. *lactis* KCTC 2013 were bought from the Korean Collection for Type Cultures (KCTC). *Lactobacillus plantarum*, and *Pediococcus acidilactici* were isolated from regular Korean yogurt. Bacterial cultures were maintained by culturing on an MRS (De Man, Rogosa and Sharpe) (Difco, Becton, Dickinson and Company, Sparks, MD, USA) agar plate. Before fermentation, bacterial culture was transferred to MRS broth medium.

### 2.4. Fermentation Experiment

The fermentation experiment was carried out in 60-mL or 120-mL glass serum bottles sealed with a rubber stopper and an aluminum cap using a Wheaton crimper (Wheaton, IL, USA). Serum bottles were filled with cucumber extract at the working volume of 50 mL. The inoculum (1%, volume/volume) was lactic acid bacteria cultivated in MRS for 24 h at 37 °C. The final cell concentration after inoculation was 3.5 × 10^5^ CFU/mL. Fermentation was performed by incubation in a shaking incubator at 37 °C and 180 rpm. During fermentation, a 1 to 2 mL sample was periodically taken and analyzed.

Initially, the original cucumber extract was directly added to the fermentation without neutralization, to test the tolerance of lactic acid bacteria in the original pH of cucumber extract. Then, fermentation by all bacteria was tested with cucumber extract neutralized to a pH of 6.5 using NaOH. The bacteria with the optimal performance were selected for fermentation of cucumber extract neutralized by HMC. The cucumber extract with initial pH 4.1 was adjusted to pH 6.5 by using 10 M NaOH or HMC slurry. As observed, HMC was not dissolved entirely when the pH reached 6.5. Approximately 11 g of HMC was required for adjusting 1 L of cucumber juice to pH 6.5. The CO_2_ gas formed during fermentation using HMC was removed by inserting needles at each sampling time. In all fermentation experiments, no additional nutrient or carbon sources were supplemented to cucumber extract.

To confirm lactic acid yield, the selected lactic acid bacteria were grown in MRS broth medium and synthetic cucumber extract. The synthetic cucumber extract was prepared by adding the relevant amount of detectable sugars and organic acids into deionized water. In addition, yeast extract, KH_2_SO_4_, MgSO_4_∙6H_2_O, and MnSO_4_∙4H_2_O at the same concentration as in the MRS medium were added to ascertain the growth of lactic acid bacteria.

### 2.5. Analytical Techniques

The pH of the cucumber extract samples was measured using a compact pH meter (LAQUAtwin-pH-22, Horiba Scientific, Kyoto, Japan). The organic acid and sugar contents of the cucumber extract were determined by high-performance liquid chromatography (HPLC) using a Dionex Ultimate3000 high-performance liquid chromatography (ThermoFisher Scientific Inc., Waltham, MA, USA). The conditions of the HPLC are summarized in Table 1. Before subjected to HPLC, all samples were centrifuged at 10,000 rpm for 5 min to remove biomass and then filtrated through a 0.2-µm syringe filter and serially diluted at the appropriate concentration for determination.

Inorganic elements, such as Mg, Na, Ca, K, Fe, V, Ni, Al, B, As, Pb, S, were analyzed using an inductively coupled plasma–atomic emission spectroscopy (ICP-AES; Activa, JY Horiba, France) at the Busan Center of the Korea Basic Science Institute (KBSI). The samples were serially diluted at an appropriate concentration before analysis.

## 3. Results and Discussion

### 3.1. Organics Content in Cucumber Extract and Pasteurization.

The bacterial enumeration of cucumber extract samples pasteurized at various temperatures and times indicated that at least 90 °C for 5 min was required to kill all indigenous bacteria in cucumber extract (Table 2). The decrease in bacteria number was correlated with the increase in temperature. In addition to temperature, the treatment time was also important for pasteurization. At the same temperature, extended treatment time resulted in a lower number of bacteria. The pasteurization at 95 °C for 1 min did not kill all bacteria, but pasteurization at 90 °C for 5 min could effectively sterilize the cucumber extract.

After pasteurization at 90 °C for 5 min, the chemical properties of cucumber extract did not change significantly (Table 3) (*p*-value < 0.05). The HPLC results indicated that the main organic components in the cucumber extract were fructose, glucose, and citric acid. Fructose and glucose were sugar sources for lactic acid fermentation. Fructose and glucose concentrations in cucumber extract were similar to those in a previous report [5], which indicated that fructose concentration was higher than glucose. Glutamic acid, tartaric acid, malic acid, and ascorbic acid are minor compounds in the cucumber extract. Ascorbic acid (vitamin C) is well-known as an antioxidant [20] with a variety of benefits to the human skin. It is interesting that this cucumber extract contains approximately 11 g/L of citric acid, which is considered as a beneficial ingredient in a magnesium gel product.

### 3.2. Fermentation of Original Cucumber Extract by Lactic Acid Bacteria

**Figure 1:**
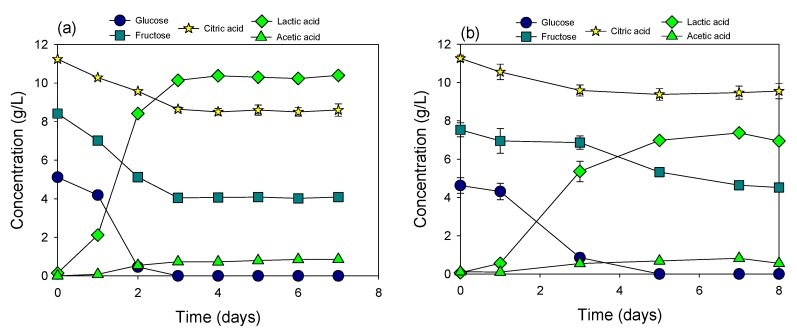
Fermentation of original cucumber extract by *Lactobacillus plantarum* (**a**) and *Pediococcus acidilactici* (**b**).

Among the investigated bacteria, only *Lactobacillus plantarum* and *Pediococcus acidilactici* could perform fermentation of the original cucumber extract at the initial pH 4.1 (Figure 1). Glucose and fructose were consumed to produce lactic acid and a small amount of acetic acid. Citric acid was slightly consumed during fermentation. Final acetic acid accumulation in both fermentation cultures was 0.7 to 0.8 g/L. Fermentation in both cultures stopped when more than half of the glucose still remained in the cucumber extract. At the time of fermentation termination, pH was dropped to 3.3 in both cultures due to the production of lactic acid. The resulting low pH killed bacteria and terminated fermentation when sugars were accumulated. Sugar contents in other cultures were stable at steady state with the incubation time extended up to 14 days (data not shown) indicating that some of the investigated bacteria were not resistant to the original pH of cucumber extract (pH 4.1).

Glucose and fructose utilization patterns in the two cultures were entirely different from each other. Both glucose and fructose were simultaneously fermented in the culture of *Lactobacillus plantarum*, whereas in the culture of *Pediococcus acidilactici*, fructose started to be consumed right after the depletion of glucose. Similar to a previous investigation [5], glucose utilization by *Lactobacillus plantarum* was slightly more rapid than fructose during the exponential fermentation phase. A higher amount of lactic acid (10.2 g/L) was produced in the culture of *Lactobacillus plantarum* compared to that (7.1 g/L) of *Pediococcus acidilactici*. However, citric acid in the culture of *Lactobacillus plantarum* was degraded more than that in the culture of *Pediococcus acidilactici.* The maximum lactic acid production in the culture of *Pediococcus acidilactici* was obtained after 7 days of fermentation, whereas it took only 3 days for *Lactobacillus plantarum* to achieve the maximum lactic acid production. Because pH was the critical factor which controlled fermentation efficiency, the cucumber extract was neutralized to improve sugar utilization and fermentation efficiency.

### 3.3. Fermentation of Cucumber Extract Neutralized by NaOH

**Figure 2:**
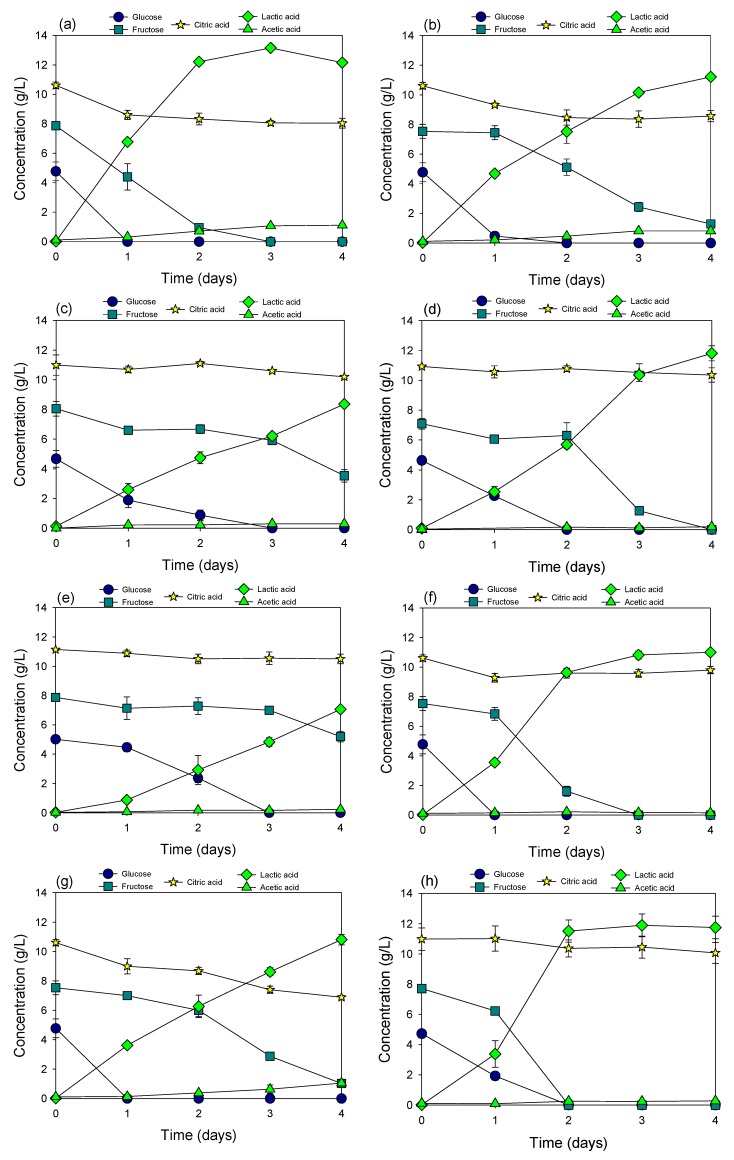

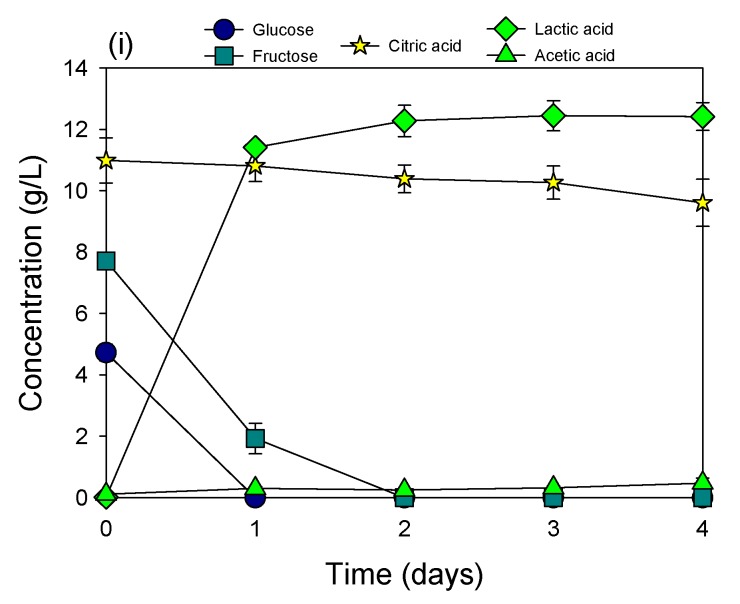
Fermentation of cucumber extract neutralized by NaOH using Lactobacillus plantarum (**a**), Pediococcus acidilactici (**b**), Lactobacillus acidophilus KCTC 3164 (**c**), Lactobacillus delbrueckii subsp. lactis KCTC 3636 (**d**), Lactococcus lactis subsp. lactis KCTC 2013 (**e**), Lactobacillus salivarius subsp. salicinius KCTC 3600 (**f**), Lactobacillus helveticus KCTC 3545 (**g**), Lactobacillus casei KCTC 13086 (**h**), and Lactobacillus paracasei KCTC 13169 (**i**).

It is clear that neutralization of cucumber extract at the initial pH 6.5 could improve fermentation efficiency in terms of both fermentation rate and sugar utilization (Figure 2). The pH of all cultures decreased to around 4.4 after 4 days of incubation, which is higher than the initial pH of cucumber extract. It took a shorter time to finish fermentation of natural sugars in cucumber juice. At pH 6.5, all investigated bacteria could perform fermentation but with different fermentation rates and sugar utilization patterns. Based on the sugar utilization patterns, the applied bacteria could be divided into two groups. The first group includes *Lactobacillus plantarum* (Figure 2a), *Lactobacillus casei* KCTC 13086 (Figure 2h), and *Lactobacillus paracasei* KCTC 13169 (Figure 2i) which are all facultatively heterofermentative lactic acid bacteria that could simultaneously utilize both glucose and fructose to produce lactic acid. The second group includes *Pediococcus acidilactici* (Figure 2b), *Lactobacillus acidophilus* KCTC 3164 (Figure 2c), *Lactobacillus delbrueckii* subsp. *lactis* KCTC 3636 (Figure 2d), *Lactococcus lactis* subsp. *lactis* KCTC 2013 (Figure 2e), *Lactobacillus salivarius* subsp. *salicinius* KCTC 3600 (Figure 2f), and *Lactobacillus helveticus* KCTC 3545 (Figure 2g), which could only utilize fructose when all glucose was depleted. The higher acetic acid accumulation in the culture of *Lactobacillus plantarum*, *Pediococcus acidilactici*, and *Lactobacillus helveticus* KCTC 3545 was correlated with the higher degradation of citric acid in these cultures, indicating that acetic acid could be a product of citric acid degradation. The transformation of citric acid to acetic acid by lactic acid bacteria has also been reported previously [21].

*Lactobacillus paracasei* KCTC 13169 represented the best bacteria in terms of lactic acid production rate. Approximately 11.5 g/L of lactic acid was produced in this culture only after 1 day of incubation. It was followed by *Lactobacillus plantarum* that produced 7.1 g/L of lactic acid after 1 day of incubation. Both these bacteria obtained the maximum lactic acid production (12.5–13.1 g/L). In addition, *Lactobacillus paracasei* KCTC 13169 could ferment all natural sugars in cucumber extract and produce 12.1 g/L of lactic acid after 2 days of incubation. Together with these three bacteria, *Pediococcus acidilactici,* which was highly resistant to low pH, was selected for the fermentation of cucumber extract neutralized by HMC.

### 3.4. Fermentation of Cucumber Extract Neutralized by HMC

**Figure 3:**
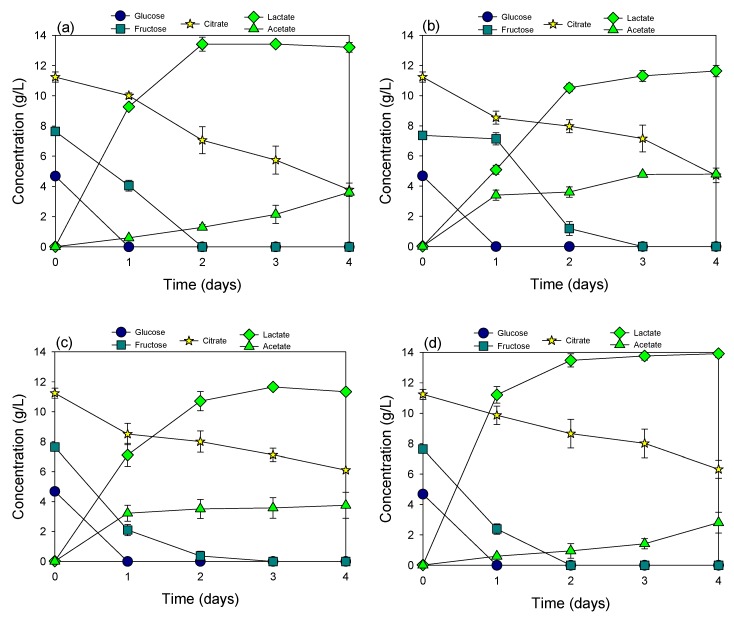
Fermentation of cucumber extract neutralized by HMC using *Lactobacillus plantarum* (**a**), *Pediococcus acidilactici* (**b**), *Lactobacillus casei* KCTC 13086 (**c**), and *Lactobacillus paracasei* KCTC 13169 (**d**).

HMC could be used as a neutralizing agent in the fermentation of cucumber extract (Figure 3). All sugars were totally consumed to produce lactic acid in all four cultures. *Lactobacillus plantarum* (Figure 2a) and *Lactobacillus paracasei* KCTC 13169 (Figure 2d) were the most efficient bacteria in this fermentation condition, which is indicated through lactic acid production and fermentation rate. Both bacteria could completely convert all sugars in cucumber extract to lactic acid within 2 days of incubation. Among them, *Lactobacillus paracasei* KCTC 13169 was slightly better than *Lactobacillus plantarum* in terms of fermentation rate. This well coincided with the result of fermentation of cucumber extract neutralized by NaOH. *Pediococcus acidilactici* (Figure 2b) and *Lactobacillus casei* KCTC 13086 (Figure 2c) required 3 days for complete consumption of all sugars, and lactic acid production was much lower than those of the other two cultures.

There was a significant difference in citric acid degradation and acetic acid accumulation in the fermentation neutralized by NaOH and HMC. Higher citric acid degradation and more acetic acid accumulation occurred in the fermentation neutralized by HMC compared to those neutralized by NaOH. This indicated that the presence of magnesium in the fermentation solution might accelerate the transformation of citric acid to acetic acid by fermenting bacteria [21], though the effect of various cations, including Na^+^, K^+^, NH_4_^+^, Ca^2+^, Mg^2+^, and Mn^2+^ on sugar utilization during cucumber juice fermentation was investigated [22]. The effect of magnesium on the transformation of citric acid in the fermentation solution is reported for the first time in this study. When lactic acid concentration reached the maximum value, the longer the incubation time was, the higher the rate of citrate breakdown into acetate. This fact was also supported by the change of the fermentation solution’s pH (data not shown). The pH of fermentation was dropped from 6.5 to 4.4 after 2 days of incubation but increased back to 5.6 after 4 days of incubation. This was the limitation of fermentation neutralized by HMC, because citric acid, which is one of the α-hydroxy acids, was proven to be a weak exfoliant and shows various benefits to human skin [23]. However, in industrial production, the fermentation could be optimized to stop right after lactic acid achieves the maximum concentration to minimize citric acid loss.

Lactic acid production from cucumber extracts neutralized by HMC was even higher than that from extracts neutralized by NaOH. Up to 13.5 g/L and 13.9 g/L of lactic acid were produced in the culture of *Lactobacillus plantarum* and *Lactobacillus paracasei* KCTC 13169, respectively, whereas the total sugar (glucose and fructose) content was only around 13 g/L. Mass balance of the fermentation system was not significantly affected by sampling because the total sample amount extracted from fermentation was tiny (<10% of total volume). Lactic acid yield in both of these cultures was higher than the maximum yield (0.98 g/g) recorded previously by a mutant strain [24]. It is possible that other minor blind compounds, such as soluble starch, in the cucumber extract could contribute to lactic acid fermentation. The transformation of soluble starch to lactic acid by *Lactobacillus* spp. was well identified in the previous study [25,26].

**Figure 4:**
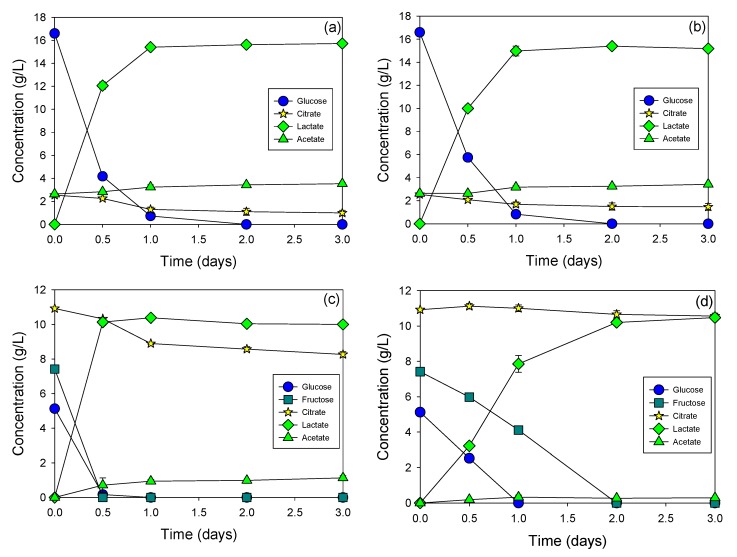
Growth of *Lactobacillus plantarum* (**a**) and *Lactobacillus paracasei* KCTC 13169 (**b**) in MRS medium and fermentation of synthetic cucumber extract by *Lactobacillus plantarum* (**c**) and *Lactobacillus paracasei* KCTC 13169 (**d**).

In fact, the growth of *Lactobacillus plantarum* and *Lactobacillus paracasei* KCTC 13169 in MRS medium, and synthetic cucumber juice again confirmed that other minor blind compounds contributed to fermentation of the pure cucumber juice (Figure 4). The lactic acid yield of *Lactobacillus plantarum* and *Lactobacillus paracasei* KCTC 13169 in the MRS medium was 0.94 and 0.93 g/g of sugar consumed, respectively. MRS medium was considered as the best broth medium for the growth of lactic acid bacteria, and lactic acid yield in this medium was always the highest. The lactic acid yield of both cultures in synthetic cucumber extract was the same (0.81 g/g sugar consumed). This was much lower than that of the MRS medium. It was interesting that the fermentation rate of *Lactobacillus plantarum* in both MRS medium and synthetic cucumber extract was slightly higher than that of *Lactobacillus paracasei* KCTC 13169, which was contrary to the fermentation patterns of pure cucumber extract. The transformation of citric acid to acetic acid by *Lactobacillus plantarum* was also higher than that by *Lactobacillus paracasei* KCTC 13169.

### 3.5. Characterization of Final Products

After 2 days of fermentation by *Lactobacillus paracasei* KCTC 13169 using HMC (12 g/L) as the neutralizing agent, the cucumber extract was introduced to the established pasteurization at 95 °C for 5 min, followed by centrifugation at 8000 rpm for 5 min to remove biomass. The final product was polished by filtering through a 0.45 µm membrane. The final product was a clear yellowish liquid. Detectable chemical contents in the final product are shown in Table 4. Compared to the original cucumber extract, glucose and fructose were depleted, and lactic acid and acetic acid were newly produced. About 27.3% of citric acid was lost due to the degradation and transformation to acetic acid. The glutamic acid concentration was not significantly changed during the fermentation. Tartaric acid and malic acid increased after fermentation, which was desirable because these hydroxyl acids also benefit the skin. As a labile compound [27], only 1.93 mg of ascorbic acid was retained in the fermented cucumber juice. After fermentation, the cucumber extract was deliberately supplemented with a significant amount of soluble magnesium ion, which could be transdermally absorbed and transferred into the bloodstream to recover aches and pains immediately. In addition to magnesium, the fermented cucumber extract also contained calcium, sodium, and potassium, which are originally present in cucumber fruit. Even though vitamin C in cucumber extract was lost during fermentation, fermented cucumber extract was compensated with a range of compounds at high concentration, which have significant value in skincare. It can be said that the skincare value of fermented cucumber extract was substantially increased compared to the original cucumber extract that is already well-known for its benefit [2,3].

## 4. Conclusions

Fermentation of cucumber extract using hydrated magnesium carbonate was successfully achieved without supplementing any additional nutrients to increase the skincare value of cucumber extract. The highest fermentation efficiency was obtained with *Lactobacillus paracasei* KCTC 13169, which can successfully convert all glucose and fructose present in cucumber extract to lactic acid. The fermentation of synthetic cucumber juice proved that there were some unknown compounds in cucumber extract that also contributed to fermentation and resulted in lactic acid production higher than that of total sugar content. The application of hydrated magnesium carbonate was limited by the transformation of citric acid to acetate. However, this can be prevented by optimizing the termination of fermentation right after all sugars are consumed. The inclusion of soluble organic acid-bound magnesium in the fermented cucumber extract at high concentration supported a novel application of this fluid as an ingredient of magnesium gels.

## Figures and Tables

**Table 1:** Conditions of high-performance liquid chromatography (HPLC) for analyzing sugars and organic acids.

Conditions	Sugars	Organic Acid
Column	YMC-Pack Polyamine II (250 × 4.6 mm)	YMC-Triart C18 (3 µm, 12 nm), 150 × 3.0 mm
Eluent	Acetonitrile/water (75/25, *v/v*)	20 mM H3PO4
Oven temp.	26 °C	37 °C
Flow rate	1.0 mL/min	1.0 mL/min
Detector	RI (Shodex RI-101, Tokyo, Japan)	UV at 220 nm
Inj. Volume	20 µL	20 µL

**Table 2:** Pasteurization test of cucumber extract.

Treatment Condition		Colony Forming Unit Per mL (CFU/mL), Mean ± S.D, n = 3
Temperature (°C)	Time (min)	
Non-treated CAF		(162.50 ± 1.41) × 10^5^
63	30	121.50 ± 4.95
72	1	119.50 ± 12.02
75	1	110.50 ± 3.5
75	5	88.00 ± 14.14
80	1	70.00 ± 7.07
80	5	12.00 ± 1.14
85	1	46.00 ± 7.07
85	5	4.00 ± 1.14
90	1	37.50 ± 4.94
90	5	0
95	1	35.50 ± 6.36
95	5	0
95	10	0
95	15	0
95	20	0
100	5	0
100	10	0
100	15	0
100	20	0
121	15	0

**Table 3:** Organic contents in cucumber extract before and after pasteurization at 90 °C for 5 min.

Cucumber Extract Types Title	Fructose (g/L)	Glucose (g/L)	Citric Acid (g/L)	Glutamic Acid (g/L)	Tartaric Acid (mg/L)	Malic Acid (mg/L)	Ascorbic Acid (mg/L)
Before	7.75	4.75	11.24	1.34	239.56	343.17	34.31
After	7.81	4.71	10.98	1.24	234.57	345.71	29.47

**Table 4:** Detectable chemical contents in cucumber extract fermented by *Lactobacillus paracasei* KCTC 13169 using HMC as the neutralizing agent.

Compounds	Chemical Content						
Organics	Citric acid (mg/L)	Lactic acid (mg/L)	Acetic acid (mg/L)	Glutamic acid (mg/L)	Tartaric acid (mg/L)	Malic acid (mg/L)	Ascorbic acid (mg/L)
	8154.1	13,921.3	1170.1	1281.2	415.00	745.12	1.93
Inorganics	Mg (mg/L)	Ca (mg/L)	Na (mg/L)	K (mg/L)	-	-	-
	3124.2	133.9	88.3	2163.6	-	-	-
